# Construction of Bouquet-like Bi_2_Se_3_/Bi_2_O_3_@Bi Composites with High Interfacial Charge Separation for the Degradation of Atrazine

**DOI:** 10.3390/ma16051896

**Published:** 2023-02-24

**Authors:** Juncheng Han, Menghan Pang, Donghuan Meng, Jianrong Qiu, Dongbo Wang

**Affiliations:** 1 School of Resources, Environment and Materials, Guangxi University, Nanning 530004, China; 2 Guangxi Universities Key Laboratory of Environmental Protection, Guangxi University, Nanning 530004, China

**Keywords:** Bi_2_Se_3_, core–shell structure, bouquet-like, atrazine

## Abstract

Using low-density solar energy in the environment and converting it into chemical energy that can drive the degradation of organic pollutants is considered to be a very promising strategy for solving the problem of environmental pollution. The efficacy of photocatalytic destruction of organic contaminants is nonetheless constrained by the high composite rate of photogenic carriers, insufficient light absorption and utilization impact, and sluggish charge transfer rate. In this work, we created a new type of heterojunction photocatalyst with a spherical Bi_2_Se_3_/Bi_2_O_3_@Bi core–shell structure and investigated its degrading properties of organic pollutants in the environment. Interestingly, benefiting from the fast electron transfer capability of the Bi^0^ electron bridge, the charge separation and transfer efficiency between Bi_2_Se_3_ and Bi_2_O_3_ is greatly improved. In this photocatalyst, Bi_2_Se_3_ not only has a photothermal effect to speed up the process of photocatalytic reaction, but also has fast electrical conductivity of topological materials at the surface, which speeds up the transmission efficiency of photogenic carriers. As expected, the removal performance of the Bi_2_Se_3_/Bi_2_O_3_@Bi photocatalyst to atrazine is 4.2 and 5.7 times higher than that of the original Bi_2_Se_3_ and Bi_2_O_3_. Meanwhile, the best samples Bi_2_Se_3_/Bi_2_O_3_@Bi showed 98.7%, 97.8%, 69.4%, 90.6%, 91.2%, 77.2%, 97.7%, and 98.9% removal of ATZ, 2,4-DCP, SMZ, KP, CIP, CBZ, OTC-HCl, and RhB, and 56.8%, 59.1%, 34.6%, 34.5%, 37.1%, 73.9%, and 78.4% mineralization. Through characterization such as XPS and electrochemical workstations, it is proved that the photocatalytic properties of Bi_2_Se_3_/Bi_2_O_3_@Bi catalysts are far superior to other materials, and a suitable photocatalytic mechanism is proposed. A novel form of bismuth-based compound photocatalyst is anticipated to be produced as a result of this research in order to address the increasingly critical problem of environmental water pollution in addition to presenting fresh avenues for the creation of adaptable nanomaterials for additional environmental applications.

## 1. Introduction

As a result of the extensive use of herbicides and antibiotics in the agricultural and medical sectors in recent years to address issues with food, clothes, and human health, trace amounts of these substances have been found in surface water, groundwater, and even in the ocean [1]. Due to their difficulty in naturally degrading in the environment, these organic contaminants build up over time and progressively endanger ecological safety [2]. To address these environmental issues, numerous studies are being conducted on the creation of effective and energy-saving methods [3]. Environmental photocatalysis, which can fully utilize low-density solar energy to destroy the majority of organic contaminants in the environment, has recently been recognized as an efficient, affordable, and environmentally friendly technology [4]. Under ultraviolet light, TiO_2_ was first demonstrated to have the ability to eliminate organic contaminants from water [5]. The practical implementation of photocatalytic technology is, however, constrained by the low utilization efficiency of sunlight and the sluggish carrier transfer rate. To address the issue of environmental organic pollution, it is necessary to create more effective and energy-efficient photocatalysts. Because of their superior photocatalytic characteristics and tunable chemical structure, Bi-based semiconductors like BiOI, Bi_2_MO_6_, Bi_2_O_2_CO_3_, and so on are of great interest to researchers [6,7,8,9].

Bismuth-based semiconductors are considered as a promising photocatalyst due to their suitable conduction and valence band positions [10]. It is worth mentioning that bismuth selenide (Bi_2_Se_3_) is a relatively special broad-spectrum responsive semiconductor, whose absorbance range can be extended to the near-infrared region due to its very narrow band gap (0.5–1.5 eV) [11]. In general, Bi_2_Se_3_ has strong topological properties and special optoelectronic properties, tha is, the material exhibits a metal surface state with a zero-band gap, allowing the presence of a large number of mobile electrons on the material surface [12]. This property may favor the transport of photogenerated electrons. However, Bi_2_Se_3_ does not show good results in the photocatalytic degradation of pollutants due to its overall high conduction and valence band positions, which leads to a weak oxidation of holes. For this reason, many researchers have made design adjustments to Bi_2_Se_3_ materials, such as the introduction of heteroatoms, construction of heterojunctions, and morphology modulation, in order to improve the photocatalytic performance of Bi_2_Se_3_. Xu et al., by co-heating previously prepared Bi_2_O_3_/g-C_3_N_4_ with selenium powder in a tube furnace, allowed the in situ reaction of Bi_2_O_3_ and Se vapor to convert to Bi_2_Se_3_, and prepared Bi_2_Se_3_/g-C_3_N_4_ photocatalyst [13]. This composite not only has a stable structure but also constitutes an S-type heterojunction, which effectively utilizes the photogenerated electrons on Bi_2_Se_3_ and increases the phenol removal efficiency of Bi_2_Se_3_/g-C_3_N_4_ by about 2.4 times compared to g-C_3_N_4_. Murugan et al. used supercritical fluid to exfoliate the bulk Bi_2_Se_3_ into Bi_2_Se_3_ nanosheets with only a few layers, and subsequently prepared exfoliated Bi_2_Se_3_ sheets/anatase TiO_2_ nanoparticles, resulting in an 80-fold increase in the hydrogen precipitation rate of the composites compared to TiO_2_, which can be attributed to the fast electron transfer and scattering effect of the topologically structured Bi_2_Se_3_ superconducting surface states [14]. Hu et al. prepared oxygen-atom-doped composites by calcining the prepared Bi_2_Se_3_ in air; the doping of oxygen atoms changed the energy band structure of Bi_2_Se_3_ and promoted the separation of photogenerated electron–hole pairs to improve the efficiency of photocatalytic removal of tetracycline [15]. Zhang et al. prepared 2D/2D Bi_2_Se_3_/g-C_3_N_4_ nanocomposite, and the combination of the two 2D materials exhibited strong built-in electric field interactions that enhanced the interfacial electron transfer capability, and the composite exhibited superior photocatalytic reduction of CO_2_ [16]. However, despite all the above-mentioned studies showing that Bi_2_Se_3_ performs well in photocatalytic applications, there are still disadvantages such as poor photocatalytic effects, complex preparation methods, and low structural stability of the composites. For this reason, we further design and modify Bi_2_Se_3_ in this paper.

Bi_2_O_3_ is a semiconductor material with a simple visible light response that is physically stable, non-toxic, and the subject of extensive research. It has been frequently demonstrated in our study group’s earlier studies that Bi_2_O_3_ and its composite materials have a positive impact on the photocatalytic destruction of pollutants [17,18]. Additionally, numerous attempts have been made to prepare Bi_2_O_3_, and the technique is now better developed. We created a Bi_2_Se_3_/Bi_2_O_3_@Bi core–shell photocatalyst for the first time, which is considerably superior than Bi_2_Se_3_ or Bi_2_O_3_ only, to accomplish efficient atrazine degradation, motivated by the aforementioned research. We simultaneously investigated its capacity for charge separation using X-ray photoelectronic spectroscopy (XPS, Thermo Scientific K-Alpha, Thermo Fisher Scientific, Waltham, MA, USA), electrochemical workstations, time-resolution photoluminescence spectra (TR-PL, FL3C-111 TCSPC, Japan), etc., and used high-performance liquid chromatography (HPLC, LC-20AD, Japan) to further reveal its capacity for mineralization, look into the environmental toxicological characteristics of degraded products, and suggest potential photocatalytic mechanisms.

## 2. Experimental Section

### 2.1. Methods

#### 2.1.1. Materials

Bismuth nitrate pentahydrate (Bi(NO_3_)_3_·2H_2_O), selenium powder (Se), ethylene glycol (EG), sodium hydroxide (NaOH), atrazine (ATZ, 98%), 2,4-dichlorophenol (2,4-DCP, 98%), sulfamethoxazole (SMZ, 98%), ketoprofen (KP, 98%), ciprofloxacin (CIP, 98%), carbamazepine (CBZ, 98%), oxytetracycline hydrochloride (OTC-HCl, 95%), and rhodamine B (RhB, AR) were purchased from Shanghai Macklin Biochemical Co., Ltd. (Shangai, China) Isopropanol (IPA, 99%), ethylenediaminetetraacetic acid (EDTA, 99%) and p-benzoquinone (p-BQ, 98%) were purchased from Sinopharm Chemical Reagent Co., Ltd. (Shangai, China). Deionized water was prepared in the laboratory. All medicines are used directly without purification.

#### 2.1.2. Preparation of Bi_2_Se_3_/Bi Nanoparticles

In general, 1.2 g of NaOH was dispersed in 30 mL of ethylene glycol, and 2 mmol of Bi(NO_3_)_3_·5H_2_O and x mmol of selenium powder (x = 1, 2, 4, 8) were added sequentially. The resulting solution was sonicated for 30 min and stirred for 60 min. Subsequently, the solution was then transferred to a stainless-steel reactor with a capacity of 50 mL of Teflon liner and placed in an oven at 180 °C for 12 h. After natural cooling to room temperature, the resulting turbid liquid was centrifuged, and the black precipitate was collected and washed several times with ethanol and deionized water. Subsequently, vacuum freeze-drying was performed and the resulting samples were recorded as nBBS (n = 0.5, 1, 2, 4).

#### 2.1.3. Preparation of Bi_2_Se_3_/Bi_2_O_3_@Bi Composite

**Scheme 1:**
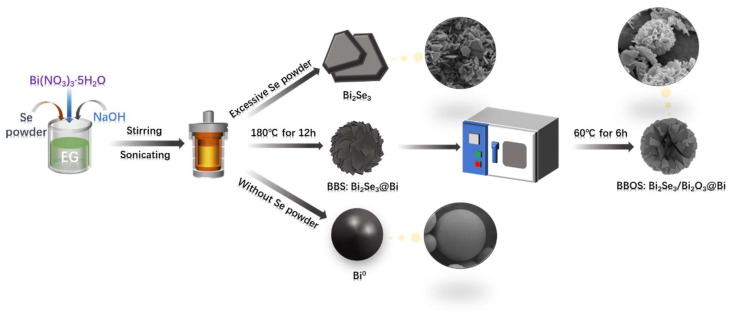
Schematic diagram for the preparation of the photocatalyst.

The above vacuum freeze-dried powder was placed in an oven and held at 60 °C for 6 h to oxidize Bi^0^ to Bi_2_O_3_, and the resulting samples were recorded as nBBOS (n = 0.5, 1, 2, 4). To explore the effect of different temperatures and times on the photocatalyst, the oven temperature and holding time were adjusted to adjust the degree of oxidation, and we set the temperature gradient T (°C) = −60, 60, 90, 120, 180, 240 at the holding time of 6 h; and the time gradient t (h) = 0, 6, 12, 18, 24 at the oven temperature of 60 °C, as shown in Scheme 1 respectively.

### 2.2. Characterization

The structure and crystallinity of the as-prepared samples were determined by X-ray diffraction (XRD, Rigaku, D/MAX 2500 V, Tokyo, Japan) analysis under the operation conditions of 40 kV and 50 mA, using Cu Kα in the range of 10°–80°. The valence states of the constituent elements were characterized by XPS (ThermoFisher Scientific K-Alpha). The molecular structure of the material is analyzed by Raman spectroscopy (inVia Reflex, Renishaw, New Mills, UK) and Fourier transform–infrared spectroscopy (FT–IR). The light absorption properties of catalysts were studied by UV-vis-NIR diffuse reflectance spectra (UV-vis DRS, SHIMADZU, UV-3600Plus, Shimadzu, Kyoto, Japan) and used to calculate the band gap of semiconductors. The photoluminescence spectra (PL) were obtained by a FL3C-111 TCSPC spectrophotometer (HORIBA, Shiga, Japan), the excitation wavelength is 380 nm. The optoelectronic properties of semiconductors were studied with an electrochemical workstation. The textural properties of the samples were analyzed by Brunauer–Emmett–Teller (BET). The microstructures of the samples were observed by scanning electron microscope (SEM) and transmission electron microscope (TEM). The oxygen vacancies and generated radicals in the samples were characterized by electron spin-resonance spectroscopy (ESR). Electrochemical measurements (Mott–Schottky, transient photocurrent, electrochemical impedance spectroscopy, and electron spin resonance) were performed using an electrochemical workstation (LK5800, China).

### 2.3. Photocatalytic Test

By testing the removal rate of ATZ (3 mg·L^–1^), 2,4-DCP (10 mg·L^–1^), SMZ (10 mg·L^–1^), KP (10 mg·L^−1^), CIP (10 mg·L^–1^), CBZ (10 mg·L^–1^), OTC-HCl (10 mg·L^–1^), and RhB (5 mg·L^–1^), the catalytic activity was evaluated. A Xenon lamp (500 W) was used to simulate sunlight exposure. The experimental methods were as follows: 50 mL pollutant aqueous solution and 50 mg photocatalyst were added into a quartz test tube (the light transmittance more than 92%), and the catalyst was evenly dispersed in the solution system by magnetic stirring. The adsorption and desorption equilibrium were tested by stirring for 60 min under dark conditions. Then the Xe-lamp was turned on, and cooling water was connected to keep the reaction temperature constant (25 °C). We tested 3 mL of solution every 20 min for its pollutant concentration. The absorbances of 2,4-DCP, SMZ, KP, CIP, RhB, and OTC-HCl were measured using a UV-vis spectrophotometer at 284 nm, 266 nm, 260 nm, 278 nm, 554 nm, and 349 nm, respectively, and the corresponding concentrations were converted according to the standard curve. The concentrations of ATZ and CBZ were determined by high performance liquid chromatography (HPLC). The pollutant removal rate was obtained by the formula $Removal\;rate=\left(1-\frac{C}{C_{0}}\right)\times 100\%$, and the total organic carbon (TOC) removal rate was obtained by the formula $Removal\;rate=\left(1-\frac{TOC}{TOC_{0}}\right)\times 100\%$. The photocatalytic stability test experiments operation was conducted as follows: the reaction liquid and the catalyst were separated by centrifugation, and the photocatalyst was washed with ethanol and deionized water several times. After vacuum freeze drying, the photocatalyst was tested again for the photocatalytic experiment. The operation procedure is consistent with the above photocatalytic experiment.

As for the radical capture experiments, photogenerated holes (h^+^), hydroxyl radicals (·OH), and superoxide radicals (·O_2_^−^) were captured using EDTA, IPA, and p-BQ, respectively. The operation procedure is consistent with the above photocatalytic experiments (test methods are available in the Supplementary Materials).

## 3. Results and Discussion

### 3.1. Material Structure Analysis

**Figure 1:**
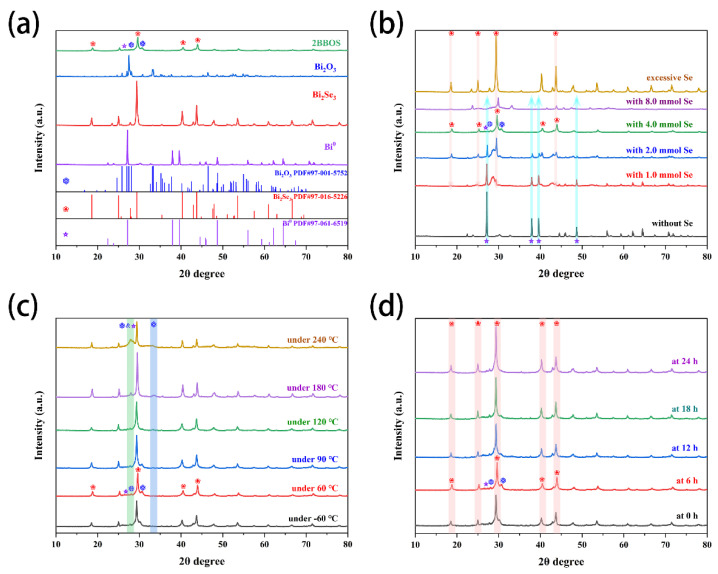
(**a**) The XRD diffractogram corresponding to Bi^0^, Bi_2_Se_3_, Bi_2_O_3_, 2BBOS, and the corresponding standard card. (**b**) The effect of adding different moles of selenium powder on BBS. (**c**) The effect of 2BBS samples treated at different temperatures for 6 h on the final product BBOS. (**d**) The effect of different times of processing of 2BBS samples at 60 °C on the BBOS of the final product (❄ corresponding to Bi_2_O_3_, ❀ corresponding to Bi_2_Se_3_, and ✰ corresponding to Bi^0^).

The content and crystal structure of the produced sample were examined using XRD. The standard cards PDF#97-001-5752, PDF#97-016-5226, and PDF#97-061-6519 correspond to the samples Bi_2_O_3_, Bi_2_Se_3_, and Bi^0^, as shown in Figure 1a. The presence of distinctive peaks for Bi_2_O_3_, Bi_2_Se_3_, and Bi^0^ at the same time in the composite material 2BBOS was discovered to be proof that the composite material was effectively manufactured [19]. In order to investigate how the amount of selenium powder added to the raw material during the synthesis of Bi_2_Se_3_@Bi affected the final products, a number of gradients characterized the final product using XRD. A tiny quantity of elemental selenium powder is left in the material after the extra selenium powder was added, as shown in Figure 1b, and practically all of the final product was converted into Bi_2_Se_3_. Bi^3+^ was converted to Bi elemental in the presence of sodium hydroxide in the ethylene glycol environment, where it finally condensed into elemental Bi^0^ spheres. Bi^0^ in the solvothermal method with the free Se in the solution created a hexagonal layer of Bi_2_Se_3_. It can be shown that the temperature of the heat treatment has a stronger impact on the composition of the material during the heat treatment operation of the Bi_2_Se_3_@Bi precursor material (Figure 1c). The peak of Bi_2_O_3_ in the sample gradually becomes more noticeable as the temperature rises. This can be attributed to the material’s oxidation of Bi^0^ to Bi^3+^ and the generation of Bi_2_O_3_ from Bi_2_Se_3_ when the oxygen in the air replaces the selenium [15]. At the same time, it was discovered that there is no discernible peak shape performance in Figure 1d, and that the effect of heat treatment time on the material’s structure and composition was not significant.

**Figure 2:**
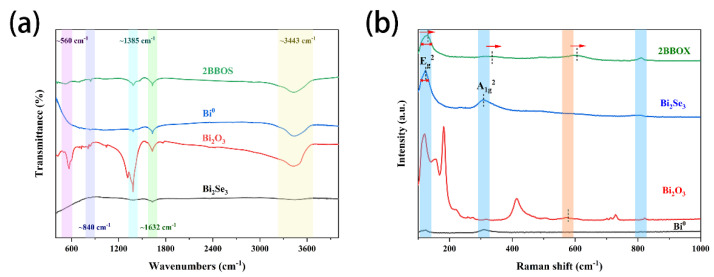
(**a**) FT–IR patterns and (**b**) Raman spectra of Bi^0^, Bi_2_Se_3_, Bi_2_O_3_ and 2BBOS samples.

The sample’s chemical bond tensile vibration mode and electron–phonon interaction were examined using Fourier transform infrared spectroscopy and Raman spectroscopy. As can be seen in Figure 2a, the peak in the sample Bi_2_O_3_ at 500–900 cm^−1^ is caused by the tensile vibration of the Bi–O link between the BiO_6_ unit and the BiO_3_ unit. The organic functional groups like –OH and –CH that have been adsorbed on the sample’s surface are responsible for the 1385 cm^−1^ and 1632 cm^−1^ peaks. Water has been adsorbed on the sample’s surface, as indicated by the signal at 3443 cm^−1^. The tensile vibration mode corresponding to Bi^0^, Bi_2_Se_3_, and Bi_2_O_3_ can be clearly seen in the composite material 2BBOS, indicating that the composite material is well prepared. In general, Bi_2_Se_3_ nanosheets comprise a five-element layer made up of five atoms of Se-Bi-Se-Bi-Se, where the Se atoms display two different chemical bond types. As seen in Figure 2b, Bi_2_Se_3_ may be seen at the two distinctive Raman peaks of E_g_^2^ and A_1g_^2^. However, in the composite material 2BBOS, the corresponding peak blue shift occurs and widens, demonstrating that during the construction of the composite material, the heterojunction changes the tensile vibration mode of the chemical bond and lattice and strengthens the electron–phonon coupling within the material [20].

### 3.2. Morphology Characterization

**Figure 3:**
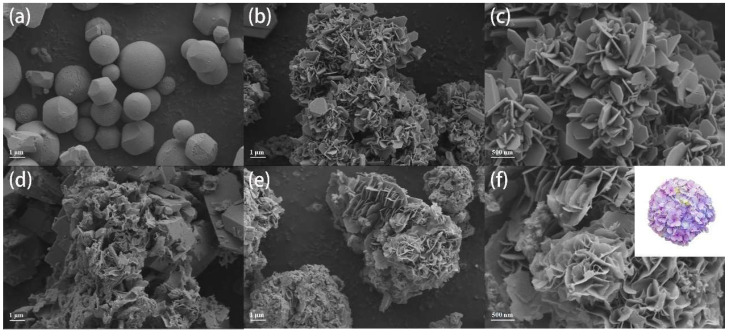
SEM images of (**a**) Bi^0^, (**b**,**c**) Bi_2_Se_3_, (**d**) Bi_2_O_3_, (**e**,**f**) 2BBOS.

SEM was used to examine the surface morphology of the material. The Bi^0^ elemental generated under alkaline circumstances has a well-dispersed spherical form, as illustrated in Figure 3a, employing ethylene glycol and Bi(NO_3_)_3_·5H_2_O as source ingredients [21]. Bi_2_Se_3_ is created when too much se powder is added during the material manufacturing process; the substance is made by an accumulation of characteristic hexagonal flakes (Figure 3b,c). An irregular flake-like Bi_2_O_3_ was created by oxidizing the Bi^0^ elemental in a humid atmosphere at 60 °C (Figure 3d). Figure 3e,f shows the final 2BBOS (Bi_2_Se_3_/Bi_2_O_3_@Bi) that has been prepared. The Bi on the surface of the Bi^0^ elemental ball is thought to react with the Se powder during the preparation procedure to create a loose sheet of Bi_2_Se_3_. The oxygen in the air combines with the Bi^0^ elemental ball that is wrapped within during a subsequent heat treatment and oxidation operation to produce Bi_2_O_3_, which ultimately creates a BBOS material. The final produced material sheet’s thickness is also thinner, which could expose more active areas and let in more light.

**Figure 4:**
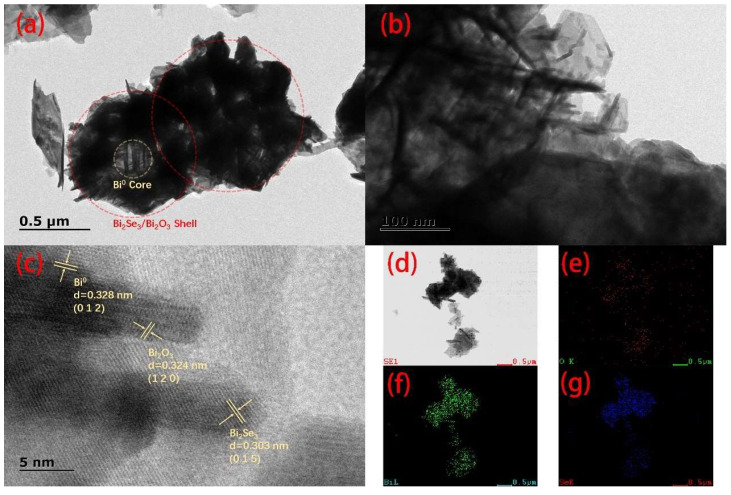
TEM image of 2BBOS: (**a**) 0.5 μm, (**b**) 100 nm, (**c**) 5 nm, and (**d**–**g**) element mapping of O, Bi, Se.

The analysis of the TEM images enabled more to be learned about the composition of the 2BBOS composite. Figure 4a demonstrates how the composite material 2BBOS has two regions, each of which has a bright core and a darker surrounding area. Generally speaking, regions of a material that have superior conductivity will appear brighter because they are more favorable for electron transmission. This demonstrates that the core–shell structure of the composite material 2BBOS, which consists of a Bi_2_Se_3_/Bi_2_O_3_ sheet layer as the shell and a Bi metal element as the core, is present. The lattice stripes corresponding to the three materials, Bi0, Bi_2_Se_3_, and Bi_2_O_3_, can be seen in Figure 4c in the HRTEM image. d = 0.328 nm corresponds to the (012) crystal surface of Bi^0^, d = 0.303 nm corresponds to the (015) crystal surface of Bi_2_Se_3_, and d = 0.324 nm corresponds to the (120) crystal surface of Bi_2_O_3_. The distribution of O, Bi, and Se elements in the composite material 2BBOS is scanned using EDS in Figure 4d,e. The simultaneous presence of three of the components O, Bi, and Se proves that the composite material was successfully put together. Additionally, the amounts of Bi elements in the substance are greater than the combined amounts of Bi elements needed to create the monomer materials Bi_2_O_3_ and Bi_2_Se_3_, confirming the presence of an elemental Bi^0^ (Figure S1).

### 3.3. Composition and Chemical State Analysis

**Figure 5:**
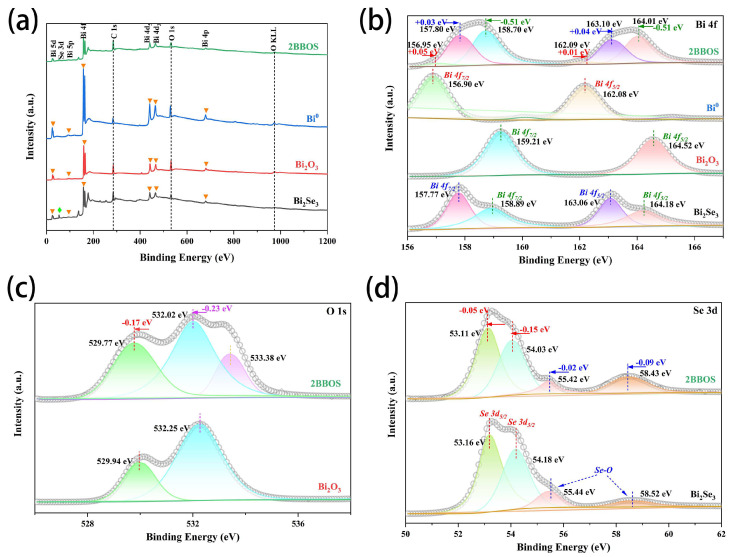
XPS spectra of Bi_2_Se_3_, Bi_2_O_3_, Bi^0^, and 2BBOS samples: (**a**) survey, (**b**) Bi 4f, (**c**) O 1s, and (**d**) Se 3d (▼ corresponding to Characteristic peaks of the Bi, ♦ corresponding to Characteristic peaks of the Se).

XPS was used to examine the sample’s chemical state in order to further ascertain the elemental composition of the prepared sample. The composite material 2BBOS is made up of Bi, Se, O, and C, according to the results of the XPS full spectrum scan of Figure 5a. The absence of distinctive peaks of other contaminants suggests that the processed substance is highly pure. Figure 5b displays the high-resolution XPS spectra of Bi 4f. The two obvious peaks of Bi_2_Se_3_, Bi_2_O_3_, and 2BBOS may be correlated with 4f_7/2_ and 4f_5/2_ of Bi^3+^, whereas the peaks of Bi^0^ can be correlated with 4f_7/2_ and 4f_5/2_ of Bi^0^, respectively, at 156.9 eV and 162.0 eV. The fact that the composite material 2BBOS moves in a direction with a high binding energy compared to the peak of Bi_2_Se_3_ and Bi^0^ at Bi 4f_7/2_ and a direction with a low binding energy compared to the peak of Bi_2_O_3_ at Bi 4f_5/2_ clearly shows that there is electron transfer between the various components of the composite material, which is helpful for creating an internal electric field. Additionally, the shape of the Bi 4f peak of the composite material 2BBOS contains and is visible with peaks corresponding to Bi^0^ and Bi^3+^, demonstrating that the preparation of the composite material is consistent with expectations [22].

The energy levels at 529.7 eV, 532.0 eV, and 533.3 eV in the high-resolution spectrum of O 1s (Figure 5c) were attributed to lattice oxygen, oxygen species adsorbed on the material’s surface, and defective oxygen. Se 3d’s high-resolution spectrum shows two peaks at 53.1 eV and 54.0 eV, which are Se 3d_5/2_ and Se 3d_3/2_, respectively. The Se–O on the surface of the material, which may have been generated by the reaction of a small quantity of imperfect selenium powder with oxygen in the air, is responsible for the peaks at 55.4 eV and 58.4 eV. There is evidence of electron transmission between the composite materials in the form of characteristic peaks in the fine spectra of O 1s and Se 3d with a minor displacement in the direction of low binding energy [23]. The aforementioned findings demonstrate that the composite material 2BBOS has been created successfully and that there is electron transfer within the substance, which may result in the formation of a heterojunction and an internal electric field.

### 3.4. Textural Properties of Catalysts

**Figure 6:**
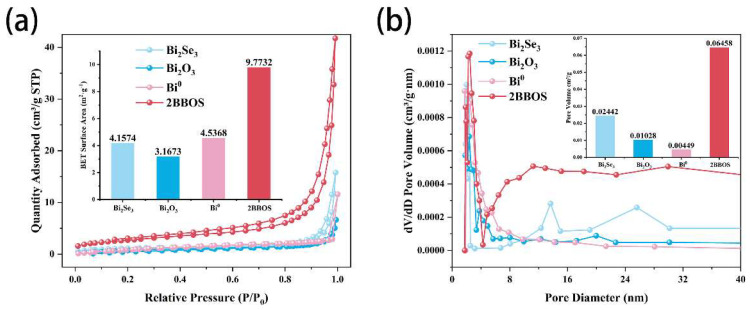
(**a**) BET N_2_ adsorption-desorption isotherms and (**b**) pore size distribution of Bi_2_Se_3_, Bi_2_O_3_, Bi^0^ and 2BBOS.

BET was used to examine the material’s pore size distribution and capacity for nitrogen adsorption and desorption. The type-IV adsorption–desorbed hysteresis curve of the 2BBOS material, as shown in Figure 6a, indicates that there are larger gaps in the sheet layer of the material, which is also visible from the SEM figure. As a result of their relatively smooth surfaces, Bi_2_Se_3_, Bi_2_O_3_, and Bi^0^ have substantially lesser adsorption capacities. The core–shell structure of 2BBOS is sheet-coated, and the composition structure is rather intricate. It has an adsorption capacity of 9.7732 m^2^/g, approximately twice as much as that of Bi_2_Se_3_ and Bi_2_O_3_. This sped up the process of surface phase breakdown of pollutants and made it easier for contaminants to adsorb on the photocatalytic surface in water bodies [24]. In addition, it is apparent that there are many more mesopores inside the composite material than there were before (Figure 6b), which is consistent with the nitrogen adsorption and desorption curve.

### 3.5. Photocatalytic Activity for ATZ Degradation

**Figure 7:**
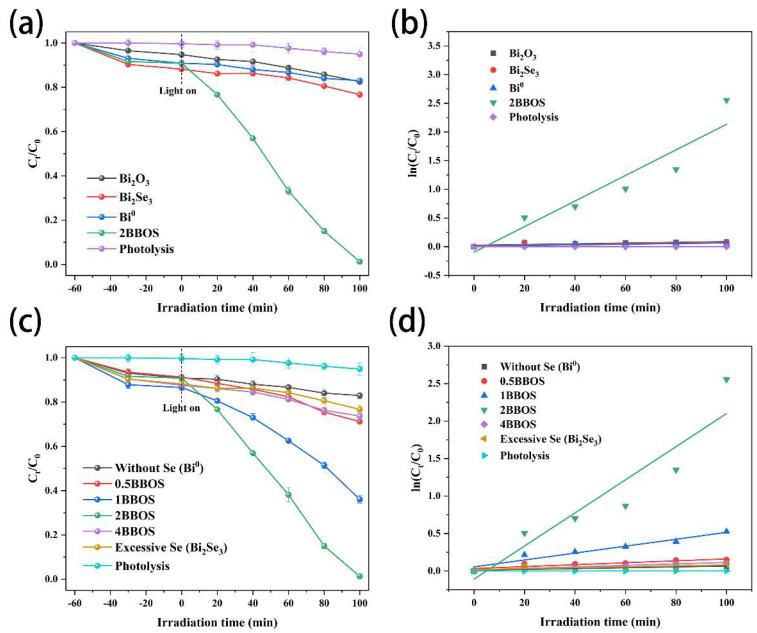
(**a**,**c**) The photocatalytic performance, (**b**,**d**) The pseudo-first-order kinetics for ATZ photocatalytic removal performance tests.

The photocatalytic characteristics of a single material and materials with various Se dosages were investigated using ATZ as the target pollutant. Bi^0^, 2BBOS, Bi_2_O_3_, and Bi_2_Se_3_ were generated separately within 100 min of light, as illustrated in Figure 7a and the removal efficiencies were 17.5%, 23.3%, 17.0%, and 98.7% on ATZ, respectively. The surface of the Bi^0^ elemental sphere in the solution was oxidized to Bi_2_O_3_ as a result of the presence of water and dissolved oxygen; therefore it has a modest photocatalytic effect that is comparable to the catalytic action of Bi_2_O_3_. Due to the quick recombination of its internal photogenic electron–hole pairs during transmission caused by the extremely narrow band gap of Bi_2_Se_3_, it is challenging to produce a nice effect. A heterojunction between Bi_2_O_3_ and Bi_2_Se_3_ can be formed, considerably increasing the photocatalytic efficiency because the constructed core–shell structure 2BBOS composite material has an appropriate energy band structure. To examine the impact of the amount of Se powder added on the photocatalytic characteristics, as seen in Figure 7c, we tried varying the amount of Se powder used. Bi^0^ elemental spheres were produced in their purest form without the addition of Se powder. Bi_2_Se_3_ was generated almost entirely when too much Se powder was added. Se powder added in excess will have an impact on the material’s performance as well as its photocatalytic capabilities, as a significant portion of the Se powder will become incomplete and remain. The photocatalytic removal efficiency of ATZ is 17.1%, 28.8%, 64.0%, 98.7%, 26.3%, and 23.3%, depending on the amount of Se powder added during the material synthesis process, which can range from 0, 0.5, 1, 2, 4 to excess. In comparison to other materials in the same series, 2BBOS composite materials have a better photocatalytic effect because different raw materials are added in the right proportions throughout the preparation process.

In order to more intuitively represent the photocatalytic efficiency of the photocatalyst sample, Pseudo-first-order reaction dynamics was used to dynamically fit the ATZ removal rate (ln(C/C_0_) = −kt, k is the rate constant) [25]. As shown in Figure 7b,d, after fitting and calculation, the reaction rate constant k of the degradation ATZ of each catalyst sample is 0.000637 (Bi_2_O_3_), 0.000608 (Bi_2_Se_3_), 0.000561 (Bi^0^), 0.00128 (0.5BBOS), 0.00461 (1BBOS), 0.0223 (2BBOS), and 0.001 (4BBOS). Among them, 2BBOS has the largest k value, and the core–shell structure 2BBOS composite material prepared with Bi^0^ as the core and Bi_2_O_3_ and Bi_2_Se_3_ as the shell has adjustable morphology and high photocatalytic activity. The drying temperature and drying time of the prepared Bi_2_Se_3_@Bi were ranked to maximize the preparation environment. As seen in Figure S2, when the drying temperature is too high and the drying time is too long, the majority of the Bi^0^ was oxidized to Bi_2_O_3_, and some of the Bi_2_Se_3_ were converted to Bi_2_O_3_, which impacts the material’s catalytic and adsorption capabilities. In the end, we found that drying at 60 °C for 6 h is the ideal preparation scenario.

**Figure 8:**
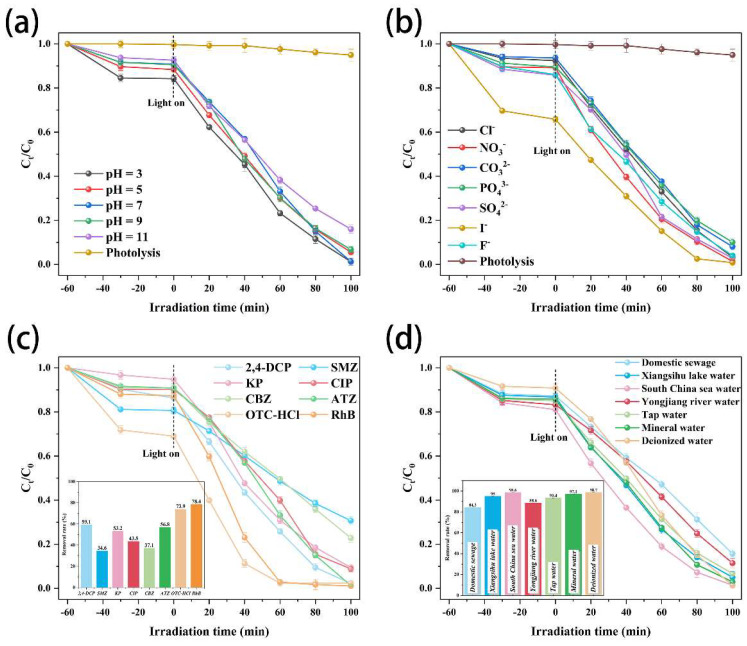
(**a**) Photocatalytic removal performance tests for ATZ by different pH and (**b**) solution containing different anions, (**c**) Photocatalytic activities of 2BBOS for 2,4-DCP, SMZ, KP, CIP, CBZ, ATZ, OTC-HCl, and RhB removal rate. (**d**) Photocatalytic removal performance tests for ATZ in different water environment.

The photocatalytic capabilities of 2BBOS composite materials in various anionic solutions and solution pH ranges were examined. Figure 8a demonstrates that 2BBOS has good photocatalytic removal efficiency for ATZ at pH 3, 5, 7, 9, and 11. The capacity of the material to adsorb pollutants diminishes significantly with an increase in pH because variations in the pH of the solution cause changes in the potential on the surface of the material. This has had an impact on the material’s photocatalytic capabilities, but they have not altered significantly and are still within acceptable limits, showing that the photocatalyst of the 2BBOS composite material can adapt to a variety of pH fluctuations in water bodies. There are typically more anions in contaminated water bodies. We set up an aqueous ATZ solution with Cl^–^, NO_3_^–^, CO_3_^2–^, PO_4_^3–^, SO_4_^2–^, I^–^, and F^–^ ions to examine the impact of various anions on the photocatalytic capabilities of 2BBOS composite materials. I^–^ ions are adsorbed on the catalyst’s surface in the photocatalytic system, as illustrated in Figure 8b, where they bind to contaminants in the water to increase the catalyst’s capacity for adsorption. Additionally, we discovered that the solution’s colorless and transparent state progressively altered during the deterioration process to one of transparency and light yellow color. It was discovered during testing with starch solution that the starch solution turned blue, indicating that some iodine ions (I^–^) were converted to iodine elemental (I_2_) during the photocatalytic process [26].

Seven organic substances as the target pollutants for catalyst universality testing and TOC removal rate testing in order to further assess the ability of the photocatalyst of the 2BBOS composite material to degrade trace pollutants. These substances were 2,4-DCP, SMZ, KP, CIP, CBZ, OTC-HCl, and RhB. The removal rate of 2BBOS from 2,4-DCP, SMZ, KP, CIP, CBZ, OTC-HCl, and RhB after 100 min of photoreaction is shown in Figure 8c to be 97.8%, 69.4%, 90.6%, 91.2%, 77.2%, 97.7%, and 98.9%, respectively. The mineralization potential of 2BBOS to organic contaminants is shown Figure 8c, insert. The mineralization rate is substantially lower than the removal rate because pollutants are mineralized into CO_2_ and H_2_O, as well as a number of small molecule intermediate products. For the composite materials 2,4-DCP, SMZ, KP, CIP, CBZ, ATZ, OTC-HCl, and RhB, the mineralization rate is 59.1%, 34.6%, 53.2%, 43.5%, 37.1%, 56.8%, 73.9%, and 78.4%. The studies demonstrate that 2BBOS composite materials have specific photocatalytic activity for various organic contaminants when compared to the prior literature (as shown Table S1).

With the prepared photocatalyst, we want to develop new techniques and technologies for the degradation of organic contaminants and apply them to the actual treatment of sewage. In order to assess the photocatalytic activity of the 2BBOS composite photocatalyst, numbers of typical surface water samples were collected to replicate a water environment. ATZ was utilized as the target pollutant. As depicted in Figure 8d, water samples from various locations were collected in the Guangxi Zhuang Autonomous Region of China, including domestic sewage in Nanning City (domestic sewage), Xiangsihu lake water in Nanning City (Xiangsihu lake water), South China sea water near the coast (South China Sea water), Yongjiang river water in Nanning City (Yongjiang river water), tap water, mountain streams in Luocheng Mulam Autonomous County, Hechi City (mineral water), and deionized water. After 100 min of light irradiation, the removal rate of ATZ in domestic sewage, Xiangsihu lake water, South China sea water, Yongjiang river water, tap water, mineral water, and deionized water of 2BBOS composite materials was 84.3%, 95.0%, 98.6%, 88.6%, 93.4%, 97.1%, and 98.7%, respectively. Reactive oxygen species were competed with ATZ in the photocatalytic process because soluble organic matter is present in household sewage, river water, and lake water. As a result, the removal rate of ATZ is lower than that of other water bodies. The elimination rate of 2BBOS to ATZ may still reach 97.1% even though the mineral water from the Guangxi karst region contains a significant quantity of Ca^2+^ and Mg^2+^ ions. This shows that high cation concentrations have no impact on photocatalysis.

The stability test of the 2BBOS photocatalytic composite materials’ photocatalytic performance to remove ATZ was also displayed at the same time in Figure S3. Its excellent photocatalytic stability may be seen by the fact that after five cycles, the removal rate of ATZ from 2BBOS can still be kept at or above 80%. XRD and SEM were used to characterize the 2BBOS samples following recycling. The chemical makeup and shape remained almost unchanged from the pictures and maps they had before use, demonstrating the photocatalyst’s superior recycling abilities for the 2BBOS [27]. By comparison with other work, 2BBOS has great potential for photocatalytic degradation of pollutants (as shown in Table S1).

### 3.6. Optical and Photoelectric Properties

**Figure 9:**
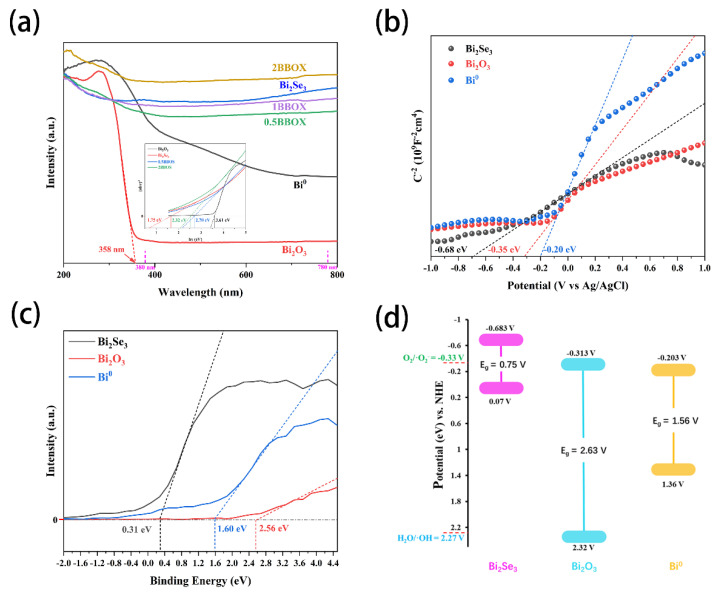
(**a**) UV-vis DRS spectra for the band gap of Bi_2_Se_3_, Bi_2_O_3_, and BBOS, (**b**) Mott-Schottky plots, (**c**) VB-XPS for valence band and (**d**) schematic band structure evolution of Bi_2_Se_3_, Bi_2_O_3_, Bi^0^, and 2BBOS.

The energy band structure and position of the material were analyzed by UV-vis DRS absorption spectrum, VB-XPS and Mott Schottky curve. As shown in Figure 9a, the UV-vis DRS absorption spectrum reflects the light absorption capacity of the synthetic material. According to previous research, Bi_2_Se_3_ has a special topology. It has a large number of structural defects inside and is almost insulated. The surface has fast conductive properties similar to metal, which results in Bi_2_Se_3_ having a very narrow band gap and exhibit properties similar to metal conductors. Therefore, it can be seen that Bi_2_Se_3_ has a strong absorption capacity of optical radiation in the range of 200–800 nm. In our previous work, it has been confirmed that the band gap of Bi_2_O_3_ is wide, and almost only ultraviolet light with shorter wavelengths can be used, and the absorption edge can only reach 358 nm [28]. Since the surface part of Bi^0^ is oxidized to Bi_2_O_3_, it is not an ideal smooth curve. Based on (αhν)^n/2^ = A(hν − E_g_) (where α is the molar absorption coefficient, h is Planck constant, ν is the incident photon frequency, and n is related to the type of semiconductor, as n = 1 for indirect transition and n = 4 for direct transition), substituting the data in UV-vis DRS, the band gaps roughly estimated of several materials, as shown in the illustration in Figure 9a. After the introduction of Bi_2_Se_3_, the band gap of the composite material 2BBOS is reduced compared to Bi_2_O_3_.

As shown in Figure 9b, the M–S curve corresponding to Bi_2_Se_3_, Bi_2_O_3_, and Bi^0^ is tangent, and the slope of the tangent is positive, which is a typical n-type semiconductor. The intersection of the tangent line and the *X*-axis is considered to be the flat potential (E_fb_) of the material. For n-type semiconductors, there is a conduction potential E_Ag/AgCl_ = E_fb_ − 0.2 V, and the potential E_CB-NHE_ = E_Ag/AgCl_ + 0.197 V relative to the standard hydrogen energy pole. Therefore, the E_CB-NHE_ corresponding to Bi_2_Se_3_, Bi_2_O_3_, and Bi^0^ are −0.683 V, −0.313 V, and −0.203 V. The valence band position of the material is analyzed by VB-XPS (Figure 9c), and the tangent of the longest smooth part of the spectral line is read (E_VB-XPS_), based on E_VB-NHE_ = φ + E_VB-XPS_ − 4.44 V (where φ is the work function of the measuring instrument), the valence band positions of Bi_2_Se_3_, Bi_2_O_3_, and Bi^0^ (E_VB-NHE_) are 0.07 V, 2.32 V, and 1.36 V, respectively. Figure 9d shows the energy band structure of several materials. The corresponding positions of Bi_2_Se_3_, Bi_2_O_3_, and Bi^0^ are shown in the figure. After being irradiated by light, ·O_2_^–^ may be produced on the conduction band of Bi_2_Se_3_, and ·OH may be produced in Bi_2_O_3_. When Bi_2_Se_3_, Bi_2_O_3_, and Bi^0^ form a composite material, it benefits from the fast electron transmission speed, which is conducive to the production of two reactive oxygen species, so that the 2BBOS composite material exhibits better photocatalytic activity [29]. This can be confirmed by free radical capture experiments.

**Figure 10:**
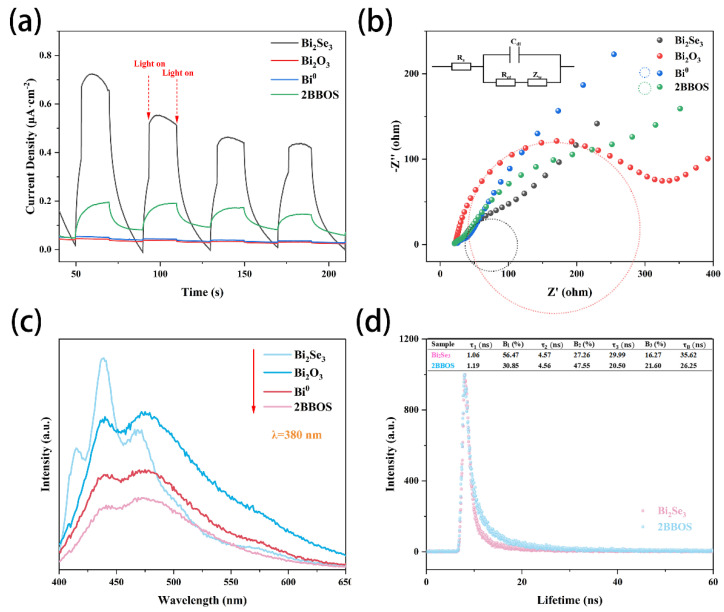
(**a**) Photocurrent responses (i-t) and (**b**) electrochemical impedance spectroscopy (EIS) curves of Bi_2_Se_3_, Bi_2_O_3_, Bi^0^ and 2BBOS. (**c**) PL and (**d**) TR-PL spectra of samples.

By using photoluminescence spectroscopy and electrochemical workstation characterization, the photoelectric properties of photocatalyst samples were examined. In Figure 10a, the transient photocurrent response spectrum is displayed. The largest peak is present in the Bi_2_Se_3_ sample, which is explained by the topological surface’s high-speed conductivity. Although it is higher than that of Bi_2_O_3_ and Bi^0^, the transient photocurrent density of the 2BBOS composite material is lower than that of Bi_2_Se_3_. This may be because the composite material’s heterojunction, which prevents the applied current from fully transmitting over the surface of Bi_2_Se_3_, forms inside the two components. The surface of Bi^0^ has a thin layer of Bi_2_O_3_ formed from a little quantity of material that has undergone oxidation, making current flow through it challenging [30]. The electrical impedance of various materials is depicted in Figure 10b. Bi_2_O_3_’s internal electrical impedance is high, as seen by the radian with a higher radius of curvature in the Nyquist diagram. The topological material Bi_2_Se_3_ has a bigger radius than 2BBOS because it has internal insulation and surface conductivity. Bi^0^ is a conductor made of metal, hence its radius is the smallest. Photoluminescence spectroscopy can measure the situation of electron–hole pairs in a material. As shown in Figure 10c, the signal of 2BBOS is weaker than that of Bi_2_Se_3_ and Bi_2_O_3_, indicating that the electron–hole pair composite can be effectively suppressed after the Bi_2_Se_3_/Bi_2_O_3_@Bi composite material is formed. It can be found that the peak value of Bi^0^ is weaker than that of Bi_2_O_3_. This is due to the fact that a small amount of the surface of the Bi^0^ elemental sphere is oxidized to Bi_2_O_3_, and it benefits from the internal Bi^0^ elemental nucleus, which accelerates the speed of electron transmission and inhibits the recombination of electron–hole pairs. In order to further analyze the electronic transport mechanism inside the material, TR-PL was used to test Bi_2_Se_3_ and 2BBOS. As shown in Figure 10d, the average life span of the carrier is determined according to the trinomial fitting. 2BBOS (26.25 ns) exhibits a shorter fluorescence life than Bi_2_Se_3_ (35.62 ns), indicating that 2BBOS has a more efficient photogenic carrier transfer rate and a faster photoelectronic generation rate. This can be attributed to the heterojunction structure inside the 2BBOS composite material and the rapid electron radiation decay pathway induced by the defect state of the topological material [31].

### 3.7. Identification of Reactive Species

**Figure 11:**
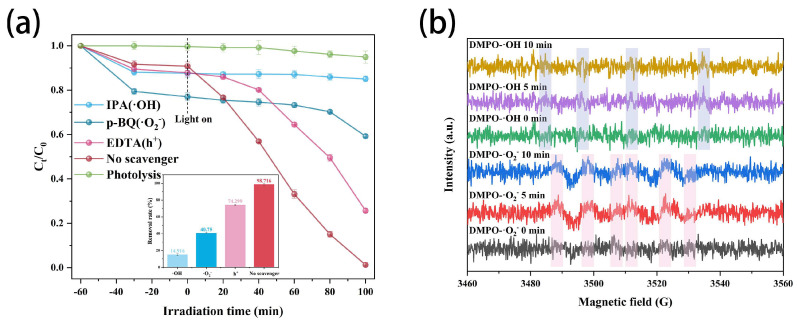
(**a**) ATZ degradation under radical capture conditions with 2BBOS. (**b**) ESR spectra of 2BBOS in Xe-lamp light.

Through free radical capture studies and ESR testing, the primary active species and mechanism of the photocatalytic reaction were investigated. In order to capture and quench the active species generated during the reaction, EDTA, IPA, and p-BQ were utilized when appropriate. As seen in Figure 11a, the photocatalytic reaction was greatly inhibited following the addition of IPA and p-BQ, whereas it was only minimally suppressed after the addition of EDTA. The contribution of several active species to photocatalytic removal of ATZ is ·OH > ·O_2_^–^ > h^+^. ·OH and ·O_2_^−^ are the main active species of the reaction. ESR detection and the usage of DMPO as a spin marker were employed to further examine the active radicals of the photocatalytic activity. Figure 11b show that after being exposed to a Xenon light for 5 min and 10 min, clear ·OH and ·O_2_^–^ signals are produced, demonstrating the presence of ·OH and ·O_2_^–^ reactive oxygen species in the reaction system [32].

### 3.8. Photocatalytic Mechanism of 2BBOS

**Figure 12:**
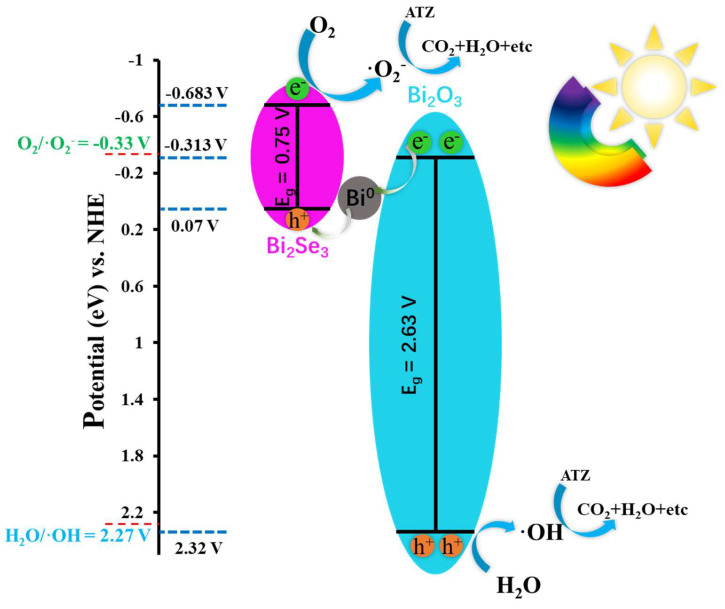
The plausible photocatalytic mechanism of the 2BBOS composite.

Based on the above experimental analysis, a photocatalytic mechanism was proposed for the degradation of organic pollutants by 2BBOS composite materials with core–shell structure. As shown in Figure 12, after being excited by light, the conduction band and valence band of the photocatalyst generated e^–^ and h^+^, respectively. The CB of Bi_2_Se_3_ is negative enough, so the e^–^ on it can be combined with the O_2_ dissolved in water to generate ·O_2_^–^, which participates in the oxidative degradation of organic pollutants [33]. The VB located on Bi_2_O_3_ is positive enough, so the h^+^ on it can react with H_2_O to form ·OH, which participates in the reduction and degradation of organic pollutants. Benefiting from the Bi^0^ metal elemental as the core of the core–shell structure, the transfer rate of photogenic electrons inside the material is greatly improved. Similar to the Z-type heterojunction structure, the electrons generated on the Bi_2_O_3_ conduction band are quickly transferred to Bi_2_Se_3_ through the Bi^0^ electron bridge and composite with the holes on it. This effectively reduces the composite of electron–hole pairs inside Bi_2_O_3_ and Bi_2_Se_3_, and greatly improves the photocatalytic efficiency [34].

## 4. Conclusions

In summary, a flower spherical Bi_2_Se_3_/Bi_2_O_3_@Bi composite photocatalyst was prepared by the solvothermal method and the drying oxidation method. The best sample 2BBOS can completely remove ATZ within 100 min, the mineralization rate can reach 61.7%, and its degradation rate constant k is about 35 times that of Bi_2_O_3_. Within 100 min, the removal rate of 2BBOS to 2,4-DCP, SMZ, KP, CIP, CBZ, OTC-HCl, and RhB reached 97.8%, 69.4%, 90.6%, 91.2%, 77.2%, 97.7%, and 98.9%, respectively, and the mineralization rate of the above pollutants was 59.1%, 34.6%, 53.2%, 43.5%, 37.1%, 73.9%, and 78.4%, respectively. In addition, cyclic experiments have shown that 2BBOS still has good photocatalytic properties after multiple uses [35]. The experimental results show that the construction of heterojunction and the Bi^0^ elemental spherical nucleus play a key role in improving photocatalytic properties. The Bi^0^ elemental spherical nucleus acts as an “electron bridge” in the heterojunction, speeding up the transfer of photogenic electrons and reducing the recombination of electron–hole pairs. The high transmission speed of electrons on the surface of the topological material Bi_2_Se_3_ improves the efficiency of producing active species ·OH, and greatly improves the performance of photocatalytic degradation of pollutants [36]. Finally, fluorescence quenching experiments and ESR were used to determine the types of photocatalytic production of active species, and proposed possible photocatalytic mechanisms.

## Data Availability

The data that support the findings of this study are available on request from the corresponding author. The data are not publicly available due to privacy or ethical restrictions.

## Supplementary Materials

The following supporting information can be downloaded at: https://www.mdpi.com/article/10.3390/ma16051896/s1, References [31,37,38,39,40,41,42,43,44,45,46,47,48,49,50,51] are cited in the Supplementary Materials.
