# Supplementary material for: Construction of Bouquet-like Bi2Se3/Bi2O3@Bi Composites with High Interfacial Charge Separation for the Degradation of Atrazine
Source: Materials (Basel). 2023 Feb 24;16(5):1896. doi: 10.3390/ma16051896 (PMC10004082; doi:10.3390/ma16051896)
Supplement: Supplementary file 1 [file materials-16-01896-s001.zip › materials-2240274-supplementary.pdf]

Supporting Information

Test methods:

To detection of reactive species of the photocatalytic elimination process, several types of scavengers were added into the photocatalytic systems under visiblelight illumination. Add EDTA, IPA, and p-BQ in the reaction system to capture photogenerated holes (h^+), hydroxyl radicals ($\cdot OH$), and superoxide radicals ($\cdot O_2^-$) separately. The dosages of all scavengers are 1 mM.

The ATZ concentration of the solution was detected by high-performance liquid chromatography (HPLC). The detection wavelength was 225 nm and the mobile phase is 70% methanol and 30% ultrapure water.

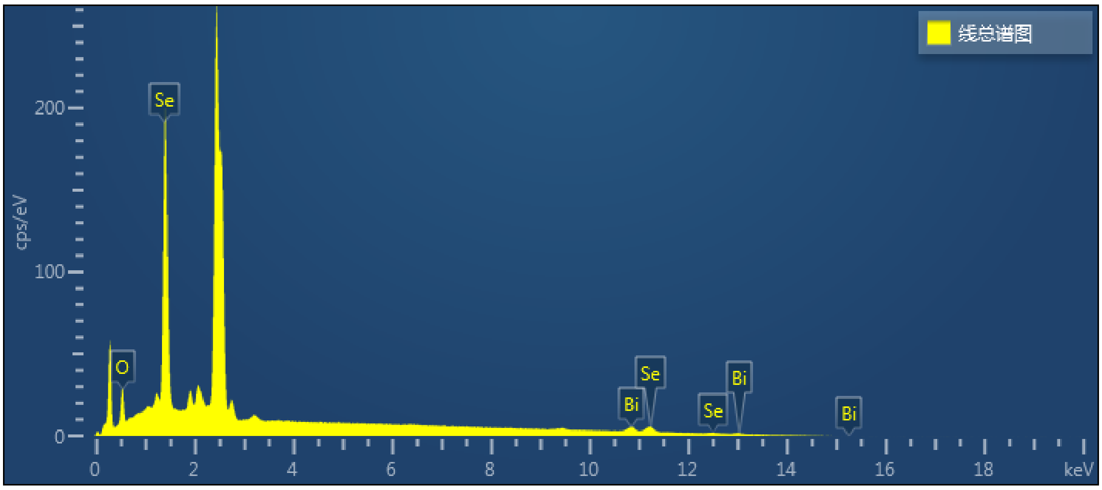

Element	Type	Weight percentage	Wt% Sigma	Atomic percentage
Se	L	23.08	0.10	31.24
Bi	M	72.16	0.12	36.91
O	K	4.77	0.07	31.85

Fig S1. EDS spectrum for 2BBOS.

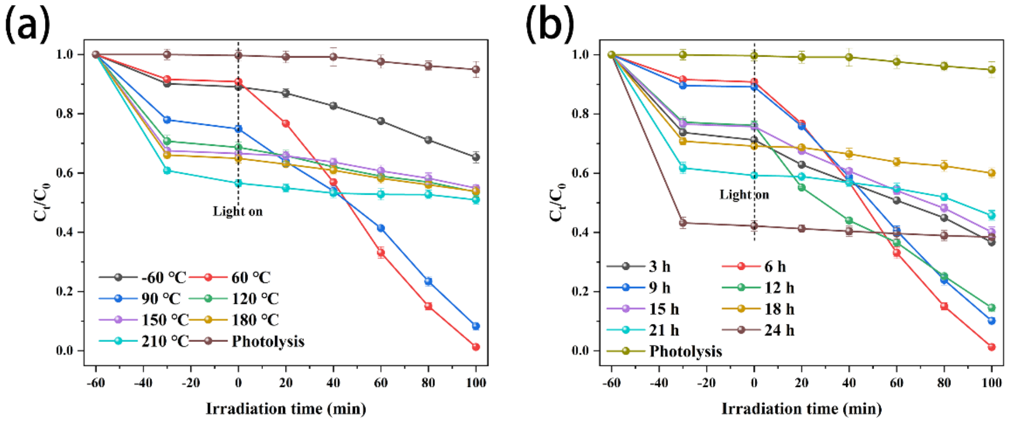

Fig S2. (a, b) The photocatalytic performance for different temperature and time.

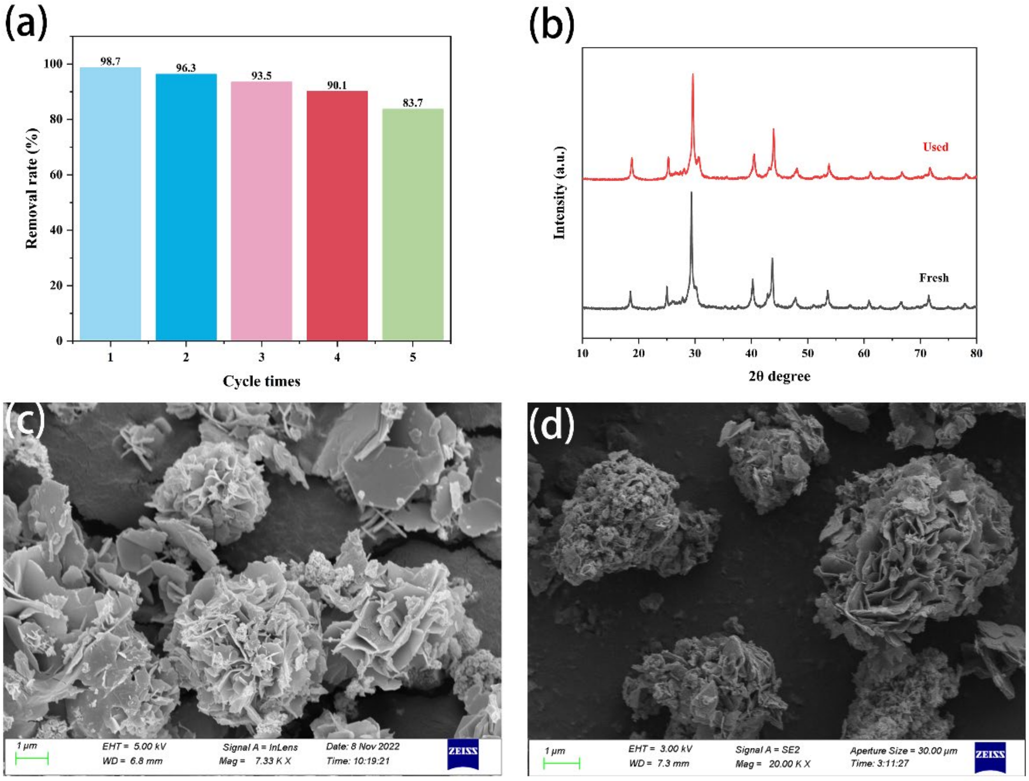

Fig S3. (a) photocatalytic stability tests. (b-d) XRD and SEM images of 2BBOS samples before and after use.

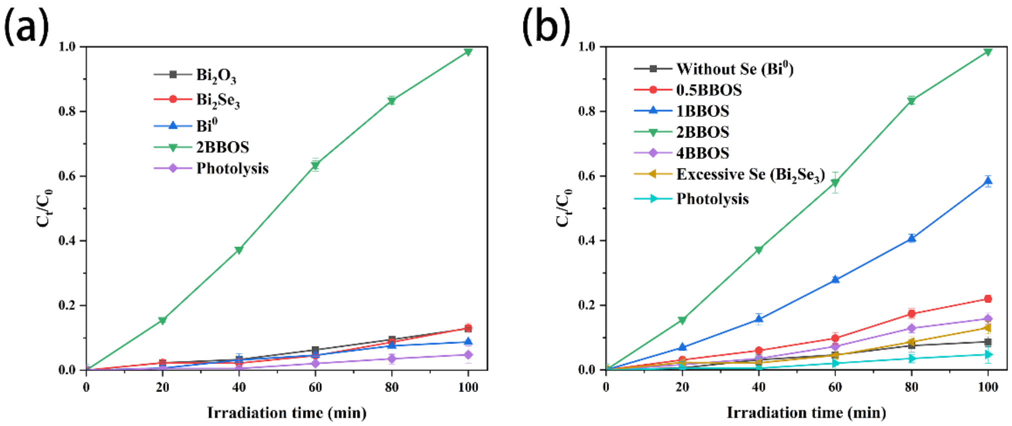

Figure S4. (a, b) The degradation efficiency of ATZ by various photocatalysts.

Table S1. Comparisons of the photocatalytic performance of several pharmaceuticals on various modified Bi_2O_3, Bi_2Se_3 in recent publications.

Pollutants	Photocatalysts	Reaction conditions				Reference (publication time)
		Photocatalysts dose (g/L)	Pollutants concentration (mg/L)	Light source (wavelength range)	Removal efficiency (%)	
Rhodamine B (RhB)	Ag/BiO_{2-x}	1	10	300 W Xe lamp (λ > 420 nm)	58.43	[1] (2022)
	$BiO_{2-x}/BiOI$	0.2	10	LED lamp	96.4	[2] (2021)
	PDI/BiO_{2-x}	0.6	5	300 W Xe lamp (λ > 400 nm)	98.7	[3] (2022)
	$BiO_{2-x}/BiOBr$	0.2	10	LED lamp	99.8	[4] (2021)
	$BiO_{2-x}/Bi_2O_{2.75}$	1	5	500 W xenon lamp (λ > 420 nm)	93	[5] (2019)
	$BiOI/BiO_{2-x}/BiOBr$	0.2	10	30 W LED lamp	99.6	[6] (2022)
Tetracycline (TC)	$Y\text{-}BiO_{2-x}$	0.1	10	300 W xenon lamp (λ > 420 nm)	74.63	[7] (2023)
	PDI/BiO_{2-x}	0.6	20	300 W Xe lamp (λ > 400 nm)	64	[3] (2022)
	$BiO_{2-x}/BiOI$	0.2	20	LED lamp	74.5	[2] (2021)
	$BiO_{2-x}/BiOBr$	0.2	20	LED lamp	79	[4] (2021)
	$BiOI/BiO_{2-x}/BiOBr$	0.2	10	30 W LED lamp	81.0	[6] (2022)
	$BiO_{2-x}/AgBiO_3$	1	20	500 W xenon lamp (λ > 420 nm)	94.1	[8] (2022)
Methyl blue	$BiO_{2-x}/AgBiO_3$	1	20	500 W xenon lamp (λ > 420 nm)	94.5	[8] (2022)

(MB)	PDI/BiO_{2-x}	0.6	5	300 W Xe lamp (λ > 400 nm)	92	[3] (2022)
	BiO_{2-x}/$NaBiO_3$	1	15	500 W xenon lamp (λ > 420 nm)	96.2	[9] (2019)
Methyl orange (MO)	PDI/BiO_{2-x}	0.6	5	300 W Xe lamp (λ > 400 nm)	88	[3] (2022)
	BiOCl/BiO_{2-x}	1	20	500 W xenon lamp (λ > 240 nm)	90	[10] (2020)
	BiO_{2-x}/$AgBiO_3$	1	20	500 W xenon lamp (λ > 420 nm)	94.1	[8] (2022)
	BiO_{2-x}/BiOI	0.2	15	LED lamp	88.7	[2] (2021)
	BiO_{2-x}/$NaBiO_3$	1	15	500 W xenon lamp (λ > 420 nm)	95.7	[9] (2019)
	BiO_{2-x}@TiO_2	1	20	500 W xenon lamp (λ > 420 nm)	91.2	[11] (2020)
Phenol	BiO_{2-x}/$NaBiO_3$	1	15	500 W xenon lamp (λ > 420 nm)	96.7	[9] (2019)
	BiOCl/BiO_{2-x}	1	20	500 W xenon lamp (λ > 240 nm)	99.4	[10] (2020)
	BiO_{2-x}-$(BiO)_2CO_3$	1	20	5W white LED light (400 nm ≤ λ ≤ 800 nm)	95	[12] (2019)
Bisphenol A	BiO_{2-x}/Bi	1	20	500 W metal halogen lamp (λ > 420 nm)	83	[13] (2018)
ATZ	PK10	0.8	10	300 W Xe lamp	83	[14] (2023)
	MI-meso-TiO_2	1	10	300 W Xe high-pressure short-arc xenon lamp	88	[15] (2019)
	$Zn_xCu_{1-x}Fe_2O_4$	1	10	500 W Xenon lamp	95	[16] (2018)

References

[1] H. Son, Y. Kim, Near-infrared driven photocatalyst (Ag/BiO_{2-x}) with post-illumination catalytic memory, Journal of Physics and Chemistry of Solids 167 (2022) 110781.

[2] L. Huang, J. Liu, Y. Li, L. Yang, C. Wang, Enhancement of photocatalytic activity of Z-scheme BiO_{2-x}/BiOI heterojunction through vacancy engineering, Applied Surface Science 555 (2021) 149665.

[3] X. Zhang, L. Shi, Y. Zhang, Preparation of organic-inorganic PDI/BiO_{2-x} photocatalyst with boosted photocatalytic performance, Journal of the Taiwan Institute of Chemical Engineers 132 (2022) 104111.

[4] J. Liu, L. Huang, Y. Li, L. Yang, C. Wang, J. Liu, Y. Song, M. Yang, H. Li, Construction of oxygen vacancy assisted Z-scheme BiO2-x/BiOBr heterojunction for LED light pollutants degradation and bacteria inactivation, J Colloid Interface Sci, 600 (2021) 344-357.

[5] M. Wang, G. Tan, D. Zhang, B. Li, L. Lv, Defect-mediated Z-scheme BiO_{2-x}/$Bi_2O_{2.75}$ photocatalyst for full spectrum solar-driven organic dyes degradation, Applied Catalysis B: Environmental 254 (2019) 98–112.

[6] J. Liu, L. Huang, Y. Li, L. Yang, C. Wang, Fabrication oxygen defect-mediated double Z-scheme BiOI/BiO_{2-x}/BiOBr photocatalyst for pollutions degradation and bacteria inactivation, Journal of Environmental Chemical Engineering 10 (2022) 106668.

[7] Y. Zhou, Q. Li, J. Zhang, M. Xiang, Y. Zhou, Broad spectrum driven Y doped BiO_{2-x} for enhanced degradation of tetracycline: Synergy between singlet oxygen and free radicals, Applied Surface Science 607 (2023) 154957.

[8] Y. Chen, Z. Han, Z. Liu, Z. Liu, P. Feng, Fabrication of flower-like BiO_{2-x}/$AgBiO_3$ photocatalysts with excellent visible light driven photocatalytic degradation, Journal of Physics and Chemistry of Solids 165 (2022) 110692.

[9] J. Wang, Z. Liu, Z. Liu, BiO2-x/NaBiO3 hybrid composites: Facile synthesis, enhanced photocatalytic activity and mechanism, Solid State Sciences, 95 (2019) 105935.

[10] Z. Liu, J. Wang, Face-to-face BiOCl/BiO2-x heterojunction composites with highly efficient charge separation and photocatalytic activity, Journal of Alloys and Compounds 832 (2020) 153771.

[11] Z. Liu, J. Xu, Fabrication of BiO_{2-x}@TiO_2 heterostructures with enhanced photocatalytic activity and stability, Applied Surface Science 511 (2020) 145460.

[12] Y. Jia, T. Zhao, D. Liu, H. Han, J. Gao, Hydrothermal synthesis of BiO_{2-x}–$(BiO)_2CO_3$ composite and their photocatalytic performance with visible light radiation, Materials Letters 238 (2019) 281–285.

[13] H. Ma, Y. Zhao, B. Souvanhthong, J. Zhao, Time-dependent synthesis of BiO2-x/Bi composites with efficient visible-light induced photocatalytic activity, J Colloid Interface Sci, 531 (2018) 311-319.

[14] Y. Deng, Z. Zhou, H. Zeng, R. Tang, L. Li, J. Wang, C. Feng, D. Gong, L. Tang, Y. Huang, Phosphorus and kalium co-doped g-C3N4 with multiple-locus synergies to degrade atrazine: Insights into the depth analysis of the generation and role of singlet oxygen, Applied Catalysis B: Environmental, 320 (2023) 121942.

[15] H. Shi, Y. Wang, C. Tang, W. Wang, M. Liu, G. Zhao, Mechanisim investigation on the enhanced and selective photoelectrochemical oxidation of atrazine on molecular imprinted mesoporous TiO2, Applied Catalysis B: Environmental, 246 (2019) 50-60.

[16] Y. Huang, C. Han, Y. Liu, M.N. Nadagouda, L. Machala, K.E. O'Shea, V.K. Sharma, D.D. Dionysiou, Degradation of atrazine by ZnxCu1−xFe2O4 nanomaterial-catalyzed sulfite under UV–vis light irradiation: Green strategy to generate SO4−, Applied Catalysis B: Environmental, 221 (2018) 380-392.
